# Treadmill Exercise Improves Motor Dysfunction and Hyperactivity of the Corticostriatal Glutamatergic Pathway in Rats with 6-OHDA-Induced Parkinson's Disease

**DOI:** 10.1155/2017/2583910

**Published:** 2017-10-24

**Authors:** Wei Chen, Decai Qiao, Xiaoli Liu, Kaixuan Shi

**Affiliations:** ^1^College of Physical Education and Sports, Beijing Normal University, Beijing 100875, China; ^2^Physical Education College, Hebei Normal University, Shijiazhuang, Hebei 050024, China

## Abstract

Hyperactivity in the corticostriatal glutamatergic pathway (CGP) induces basal ganglia dysfunction, contributing to parkinsonian syndrome (PS). Physical exercise can improve PS. However, the effect of exercise on the CGP, and whether this pathway is involved in the improvement of PS, remains unclear. Parkinson's disease (PD) was induced in rats by 6-hydroxydopamine injection into the right medial forebrain bundle. Motor function was assessed using the cylinder test. Striatal neuron (SN) spontaneous and evoked firing activity was recorded, and the expression levels of Cav1.3 and CaMKII in the striatum were measured after 4 weeks of treadmill exercise. The motor function in PD rats was improved by treadmill exercise. SN showed significantly enhanced excitability, and treadmill exercise reduced SN excitability in PD rats. In addition, firing activity was evoked in SNs by stimulation of the primary motor cortex, and SNs exhibited significantly decreased stimulus threshold, increased firing rates, and reduced latency. The expression of Cav1.3 and p-CaMKII (Thr286) in the striatum were enhanced in PD rats. However, these effects were reversed by treadmill exercise. These findings suggest that treadmill exercise inhibits CGP hyperactivity in PD rats, which may be related to improvement of PS.

## 1. Introduction

The pathological basis of Parkinson's disease (PD) is the degeneration of dopaminergic neurons in the substantia nigra pars reticulata, which leads to a substantial loss of dopamine (DA) in the striatum and results in motor control dysfunction in the basal ganglia [1]. Dopamine depletion results in hyperactivity of the corticostriatal glutamatergic pathway, along with corresponding changes in the direct and indirect pathways of the basal ganglia; this is thought to be responsible for parkinsonian symptoms [1, 2]. Indeed, *in vitro* electrophysiological studies have suggested that striatal spiny neurons exhibit a significant increase in glutamate-mediated spontaneous synaptic events after dopaminergic neurons are damaged by 6-hydroxydopamine (6-OHDA) injection [2]. This may be related to increased levels and release of striatal glutamate from corticostriatal terminals [3, 4]. Therefore, clinical parkinsonian syndrome, especially motor dysfunction, may be associated with hyperactive corticostriatal glutamatergic transmission [4, 5].

The beneficial effects of physical activity on motor function of patients with PD have been suggested previously [6, 7]. In animal studies, exercise has also been reported to reduce glutamatergic synaptic activity, decrease striatal glutamate levels, and improve motor dysfunction [8–10]. These studies indirectly supported the link between improved motor dysfunction and reduced corticostriatal glutamatergic activity. However, no study has yet reported the effect of exercise on corticostriatal glutamatergic pathway excitability in PD. To investigate the neurobiological mechanisms of the effect of exercise on basal ganglia dysfunction in PD, we recorded spontaneous firing activity from striatal neurons using electrophysiological techniques and assessed the firing activity evoked in striatal neurons by stimulating the primary motor cortex (M1) in PD model rats.

## 2. Materials and Methods

### 2.1. Experimental Subjects

Male Sprague−Dawley rats weighing 220−240 g (8 weeks of age) were obtained from Beijing HFK Bioscience Co. Ltd. (Beijing, China) and were randomised into 4 groups: (1) control group (control, *n* = 50), (2) control plus exercise group (control + Ex, *n* = 50), (3) Parkinson's disease group (PD, *n* = 90), and (4) Parkinson's disease plus exercise group (PD + Ex, *n* = 90).

All experiments were carried out with the approval of the Life Sciences School Animal Ethics Committee of Beijing Normal University. Rats were group-housed at a stable temperature (22−25°C) and air humidity (45−50%) under a 12 h light/dark schedule with ad libitum access to food and water.

### 2.2. Establishment and Evaluation of the Rat Model of Parkinson's Disease

**Figure 1:**
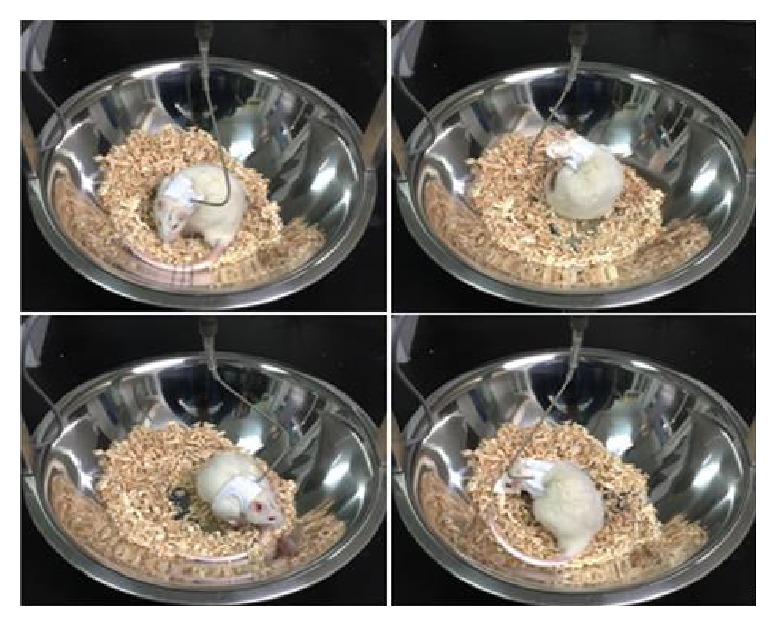
Apomorphine- (APO-) induced rotational behaviour in Parkinson's disease (PD) model rats. The striatal dopamine receptors can be super-sensitized due to a marked decrease in striatal dopamine concentration by 6-OHDA injection. Therefore, apomorphine, which is a dopamine receptor agonist, injected into the peripheral tissues, can induce the rats to exhibit rotational behaviour to the left.

A solution of 2 *μ*g/*μ*L 6-OHDA (Sigma, St Louis, MI) was prepared from 6-OHDA and saline containing 0.02% ascorbic acid (Sigma). The PD and PD + Ex rats were anaesthetised with chloral hydrate solution (3.5 mL/kg, Sigma), fixed onto a stereotaxic apparatus (RWD, Shenzhen, China), and injected with 4 *μ*L of the 6-OHDA solution at a rate of 1.0 mm/min into the right medial forebrain bundle (MFB; AP: −4.3 mm, R: 1.5 mm, V: 7.6 mm). The rats in the control and control + Ex groups were injected with saline. After surgery, all rats were treated with benzathine penicillin (300,000 IU/kg) to prevent infection. The PD rats were evaluated 7 days after the unilateral 6-OHDA injection in terms of rotational behaviour (turns/min) induced by subcutaneous injection of apomorphine (0.5 mg/kg, Sigma) into the neck [11]. The rotation numbers to the left were recorded 3 min after apomorphine injection, and rats making at least 100 turns/30 min were selected for use in the present study [12], as shown in Figure 1.

To estimate the injury of dopaminergic neurons in the substantia nigra of PD rats, the expression of tyrosine hydroxylase (TH) was detected by immunohistochemical methods. Seven days after surgery, 6 rats were randomly selected from the PD model rats; the number of APO-induced rotational laps in these rats was in accordance with the standard of the PD model and the rats in the sham operation group, respectively. Rats were anaesthetised with 10% chloral hydrate solution (3.5 mL/kg) and perfused with 37°C saline, followed by 4% paraformaldehyde, through the left ventricular-ascending aorta; the brain was then quickly removed and placed in 4% paraformaldehyde. TH immunostaining was performed on rat substantia nigra sections as previously described [8]. Briefly, brain tissue slices were rinsed in 0.1 M phosphate buffer, incubated with 3% H_2_O_2_, blocked with 10% serum albumin and 0.3% Triton X-100 in 0.1 M phosphate buffer, and then incubated with rabbit anti-TH antibody (Abcam, Cambridge, MA; 1 : 500) overnight at 4°C. Brain tissue slices were rinsed in 0.1 M PB and incubated with horseradish peroxidase-labelled goat anti-rabbit IgG (ZHGB, 1 : 200) at 37°C for 30 min, followed by rinsing, DAB staining, air-drying, vitrification with dimethylbenzene, and sealing with neutral gum. Substantia nigra images were collected under an imaging microscope (Olympus, BX61-DP72, Tokyo, Japan). The average optical density of TH-positive cells was analysed using Image-Pro Plus 6.0 software (Media Cybernetics Inc., Bethesda, ML).

### 2.3. Exercise Intervention Program

Twenty-four hours after creating the 6-OHDA lesion, the rats in the treadmill exercise groups (control + Ex and PD + Ex) were forced to run on a motorized treadmill (Duanshi, Hangzhou, China) for 30 min/day at a speed of 11 m/min, for 4 consecutive weeks. Rats in the nonexercise groups (control and PD) were left in the treadmill without running for the same duration of time.

### 2.4. Behavioural Testing

**Figure 2:**
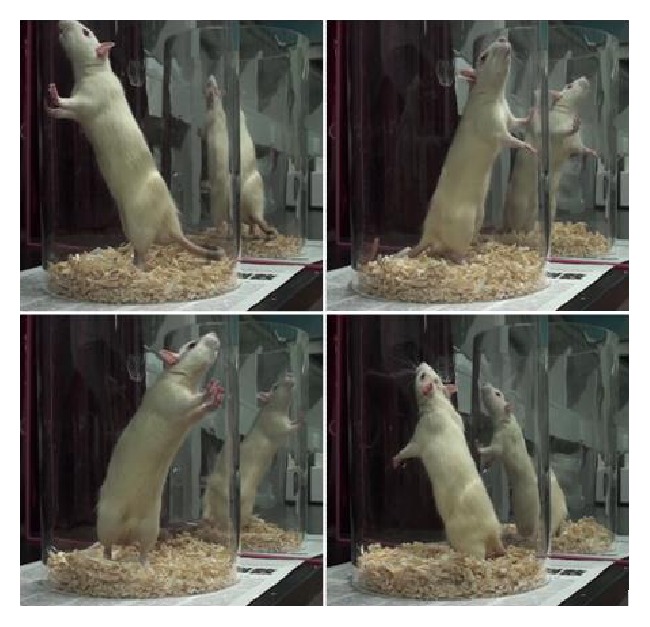
The cylinder test. Contacts were recorded if the rat contacted the cylinder wall once with a foreleg unilaterally, or kept this foreleg in contact with the cylinder wall, and then touched the cylinder wall with another leg, or contacted the cylinder wall with both forelegs at the same time.

The cylinder test is mainly used for evaluation of the asymmetry of forelimb activity. At 7, 14, and 28 days after creating the 6-OHDA lesion, the rats were placed in a transparent Plexiglas cylinder (20 × 30 cm) with mirrors placed behind the cylinder to observe the rats fully; the number of contacts of the forelimbs with the cylinder wall was recorded for 3 minutes using a high-definition camera. The formula for calculating the ratio of the left foreleg contacts with the cylinder wall was taken from Karhunen et al. [13], as shown in Figure 2.

(1) $$\begin{array}{c} The\;ratio\;of\;the\;left\;foreleg\;contacts\;with\;cylinder\;wall=\frac{a+c\times 0.5}{a+b+d}\times 100\%, \end{array}$$

where *a* is the number of left forelimb contacts with the cylinder wall, *b* is the number of bilateral forelegs contacts with the cylinder wall, *c* is the number of simultaneous bilateral foreleg contacts with the cylinder wall, and *d* is the number of right forelimb contacts with the cylinder wall.

### 2.5. Electrophysiological Recording

Twenty-four hours after the last treadmill exercise session, 12 rats were randomly selected from each group; rats were anaesthetised with chloral hydrate solution (3.5 mL/kg, i.p.) and fixed in a stereotaxic apparatus (RWD, Shenzhen, China). Striatal neuron firing activity was recorded using glass microelectrodes with tip diameters of 3−5 *μ*m and impedance of 3−8 M*Ω*, and filled with a 0.9% sodium chloride solution. The glass microelectrode was inserted into the dorsal striatum (AP: −1.0 to 1.0, R: 3.0 to 4.5, and DV: 3.5 to 6.0) with a microelectrode manipulator under a dissecting microscope, according to the rat brain atlas (Paxinos, 1997). Electrical signals were inputted to the Powerlab Biological Signal Processing System via an amplifier (2400, AD Instrument, Colorado Springs, CO) and analysed using Labchart 7.0 software (AD instruments, Bella Vista, Australia).

### 2.6. Cortical Electric Stimulation

**Figure 3:**
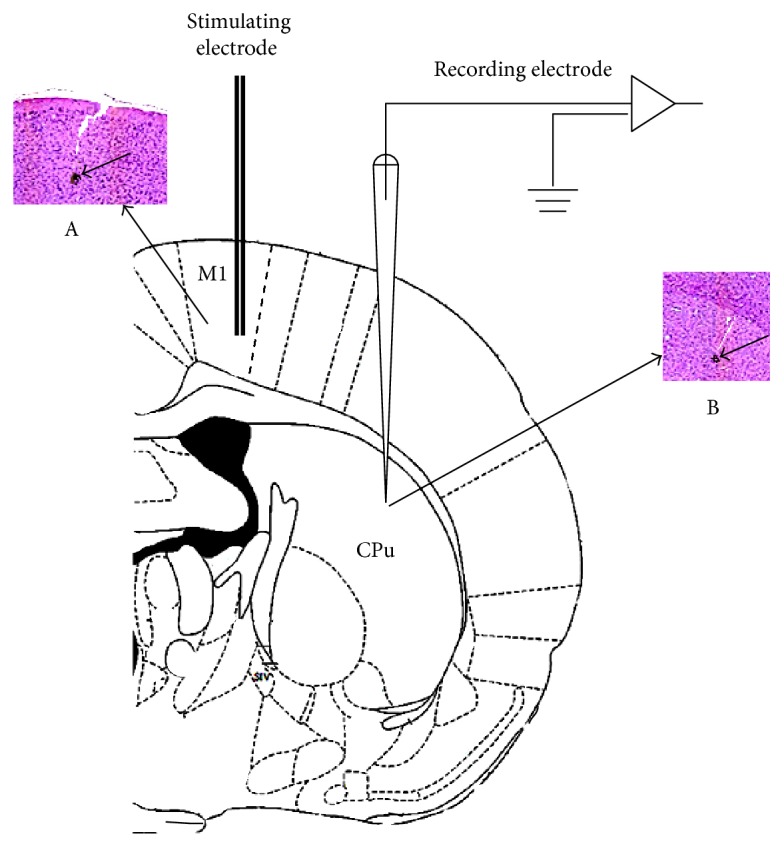
Schematic diagram of the positions of the recording electrodes and stimulating electrodes in the rat brain. M1: primary motor cortex. CPu: caudate putamen. A: haemorrhagic spots induced by stimulating electrode fulgerization located in the M1 region. B: haemorrhagic spots induced by recording electrode fulgerization located in the CPu region.

The primary motor cortex (M1) was stimulated with tungsten wire bipolar electrodes through a stainless steel tube (inner diameter: 300 *μ*m), with an insulating layer on the surface. Stimulating electrodes were implanted among the pyramidal cells of layer V of the primary motor cortex and were activated with a microelectrode manipulator. The intensity of the stimulus was gradually increased until striatal neuron firing activity was evoked and the threshold stimulus intensity was recorded. Then, the intensity of the stimulus was stabilised at 1.5 mA; the wave width was 0.1 ms, the delay time was 5 ms, the repetition frequency was 5 times, the main period was 1 s, and the electrical signals were continuously collected for 15 min, as shown in Figure 3.

### 2.7. Western Blot Analysis

Expression of Cav1.3, CaMKII, and p-CaMKII (Thr286) in the striatum was detected using Western blot analysis. Six rats were randomly selected from every group. Frozen brain tissues were subjected to coronal sectioning; the striatum was obtained, ground finely, and an appropriate amount of lysate buffer was added. Lysis was allowed to proceed for 30 min, and the solution was then centrifuged at 12000 r/min for 10 min. The supernatant was obtained and aliquoted, and protein concentrations were measured using a bicinchoninic acid assay. Then, the samples were electrophoresed on polyacrylamide gels and transferred to polyvinylidene difluoride (PVDF) membranes. Membranes were blocked and incubated with diluted monoclonal antibodies (Abcam, Cav1.3: 1/500; CaMKII: 1/200; p-CaMKII: 1/1000) overnight. HRP-labelled secondary antibodies were added and the samples were incubated for 60 min. Bands were detected using enhanced chemiluminescence (ECL). After exposure, membranes were rinsed and blocked. *β*-Actin monoclonal antibody was diluted 1 : 5000 in primary antibody dilution buffer, and gently shaken at room temperature for 60 min. The internal reference band was obtained by ECL. Gel image processing software was utilised to analyse the molecular weight and integral optical density of the target bands.

### 2.8. Data Analysis

The electrical signals of the striatal neurons were analysed using Labchart 7.0 software. One-way ANOVA and Tukey's post hoc comparisons were used to analyse the electrophysiological, biochemical, and behavioural variables between groups. Paired-samples *t*-test comparisons of discharge frequency of the striatal neurons before and after cortical stimulation were performed. Chi-square tests were used for analysis of the firing mode and frequency distribution of striatal neurons between groups. Values are given as means ± SEM. The criterion for significance was set at *P* < 0.05.

## 3. Results

### 3.1. Treadmill Exercise Improves Forelimb Motor Dysfunction in PD Rats

To assess the effect of exercise on motor function in PD rats, we tested forelimb-use asymmetry by cylinder test.  Table 1 shows that left forelimb asymmetry scores of both PD and PD + Ex rats were decreased at 7, 14, and 28 days after the 6-OHDA-induced lesioning compared to those in the control group (*P* < 0.01), indicating a tendency of 6-OHDA-lesioned rats to use the right forelimb. However, there was no difference in this variable between PD and PD + Ex rats (*P* > 0.01) at 7 and 14 days after the 6-OHDA-induced lesioning, but this difference was significant at 28 days after the 6-OHDA-induced lesioning (*P* < 0.05). This suggests that exercise can improve motor function in PD rats, but the effect of exercise needs to accumulate to some extent.

**Figure 4:**
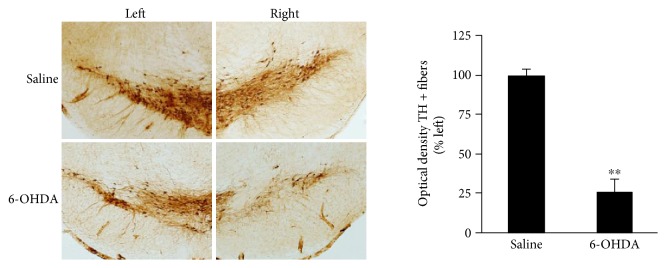
Expression of tyrosine hydroxylase (TH) in the substantia nigra of rats 7 days after saline (control and control + Ex) and 6-OHDA (PD and PD + Ex) injection. Note that expression of TH in the substantia nigra was observed at 7 days after 6-OHDA injection in rats checked randomly. Compared to the saline group, ^∗∗^*P* < 0.01. Control: control group; control + Ex: control plus exercise group; PD: Parkinson's disease group; PD + Ex: Parkinson's disease plus exercise group; 6-OHDA: 6-hydroxydopamine.

To investigate the loss of dopaminergic neurons in rats in which the number of APO-induced rotational laps was in accordance with the standard of PD model, TH immunohistochemical analysis of the substantia nigra was performed 7 days after the unilateral 6-OHDA injection, in order to test the reliability of the PD animal model. We found that the expression of TH was significantly decreased in the 6-OHDA injection group compared to the expression in the saline injection group (*P* < 0.01), suggesting that the PD rat model was successfully established in the present study, as shown in Figure 4.

### 3.2. Analysis of the Firing Pattern of Striatal Neurons

**Figure 5:**
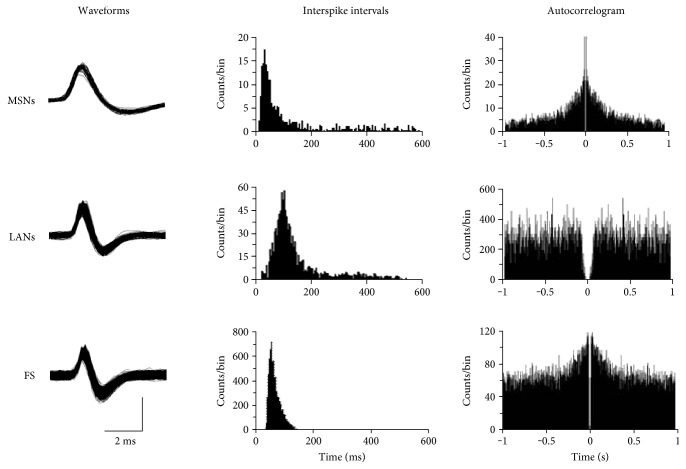
The spontaneous firing patterns of striatal neurons. Typical action potential waveforms (left column), interspike intervals (middle column), and autocorrelograms (right column) associated with medium spiny-like neurons (MSNs), large aspiny-like neurons (LANs), and fast-spiking interneurons (FS).

**Figure 6:**
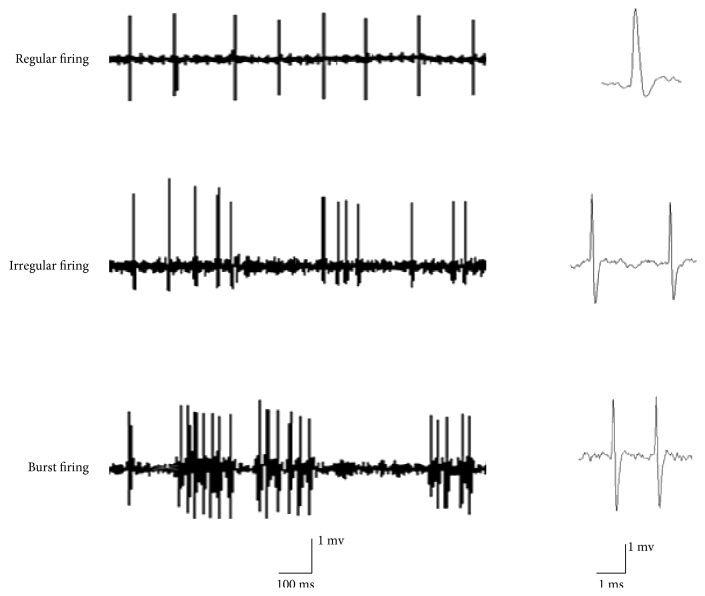
Different neuronal firing patterns in the striatum. Regular firing pattern: ISI histogram exhibiting a symmetrical distribution. Irregular firing pattern: ISI histogram exhibiting a random distribution. Burst firing pattern: ISI histogram exhibiting an obvious positive skewness with a long progressive decline.

In the present study, 58 striatal neurons were recorded in the control group, and 75, 116, and 98 neurons were recorded in the control + Ex, PD, and PD + Ex groups, respectively. According to the characteristics of the interspike interval histogram of the striatal neurons, spontaneous firing activity was divided into three forms: regular firing, irregular firing, and burst firing, as shown in Figures 5 and 6.

### 3.3. Treadmill Exercise Reduces Spontaneous Firing Activity of Striatal Neurons in PD Rats

**Figure 7:**
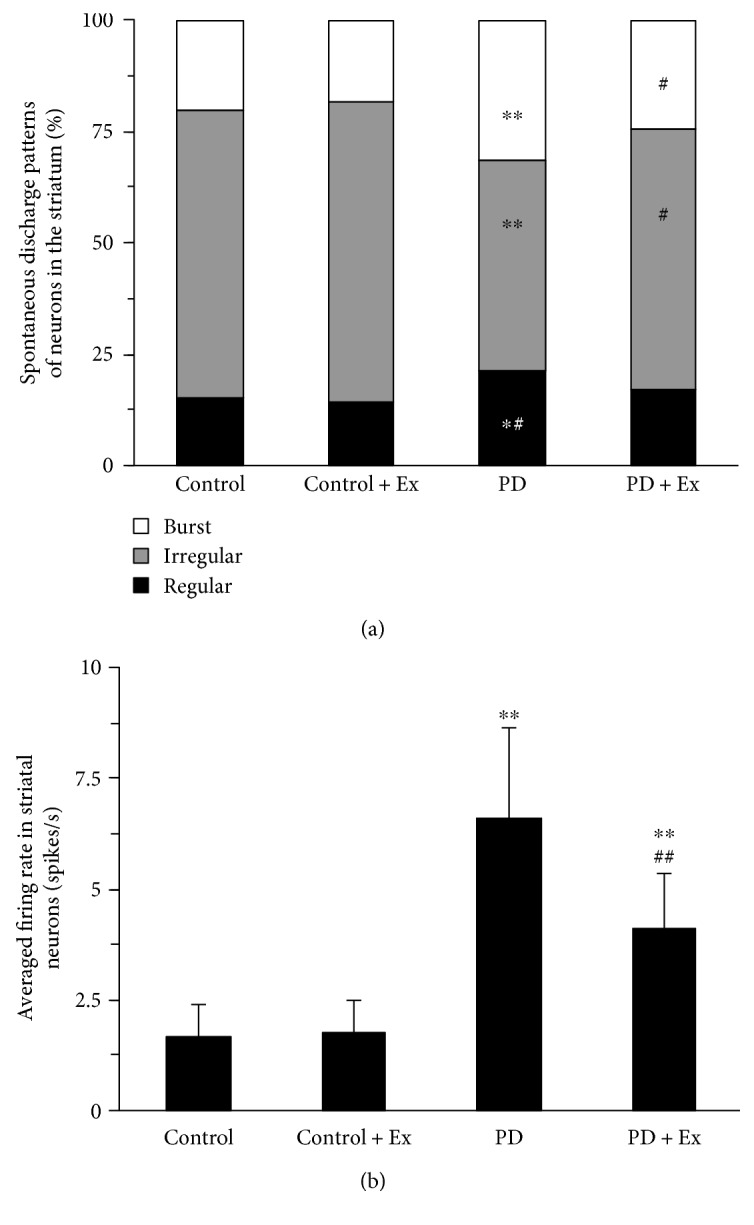
Effect of treadmill exercise on spontaneous firing patterns and average firing rate of striatal neurons in the rat model of Parkinson's disease. (a) Analysis of spontaneous firing patterns in striatal neurons. (b) Change in the average firing frequency in striatal neurons. Compared to the control groups, ^∗^*P* < 0.05 and ^∗∗^*P* < 0.01. Compare to the PD groups, ^#^*P* < 0.05 and ^##^*P* < 0.01. Control: control group; control + Ex: control plus exercise group; PD: Parkinson's disease group; PD + Ex: Parkinson's disease plus exercise group.

A total of 58 striatal neurons were recorded in control rats, and 75, 116, and 98 striatal neurons were recorded in control + Ex, PD, and PD + Ex rats, respectively. Spontaneous firing activity of striatal neurons, according to the characteristics of the interspike intervals, was divided into three forms: regular discharge, irregular discharge, and burst discharge.  Figure 7(a) shows that PD rats had more burst (31.8% versus 19.6%) and regular (21.7% versus 15.1%) firing neurons than the control group (*P* < 0.01 and *P* < 0.05, resp.). There were significantly fewer irregularly firing neurons (47.4% versus 64.3%) in the PD than in the control group (*P* < 0.01). Moreover, there were significantly fewer burst firing neurons (24.2% versus 31.8%) and significantly more irregularly firing neurons (58.3% versus 47.4%) in the PD + Ex than in the PD group (*P* < 0.05 and *P* < 0.05, resp.). This suggested that treadmill exercise has an inhibitory effect on the spontaneous firing activity of striatal neurons in 6-OHDA-induced PD rats.

Figure 7(b) shows that PD rats (6.62 ± 2.01 Hz) and PD + Ex rats (4.13 ± 1.24 Hz) had greater average striatal neuron firing rates than those of control rats (1.71 ± 0.67 Hz) rats (*P* < 0.01 and *P* < 0.01, resp.). Furthermore, the rats in the PD + Ex group had lower average firing rates than the PD group (*P* < 0.01), suggesting that treadmill exercise may reduce the firing rates of striatal neurons in PD rats.  Table 2 shows changes in striatal neuron firing rates for each group.

### 3.4. Treadmill Exercise Decreases the Firing of Striatal Neurons Evoked by Cortical Stimulation in PD Rats

**Figure 8:**
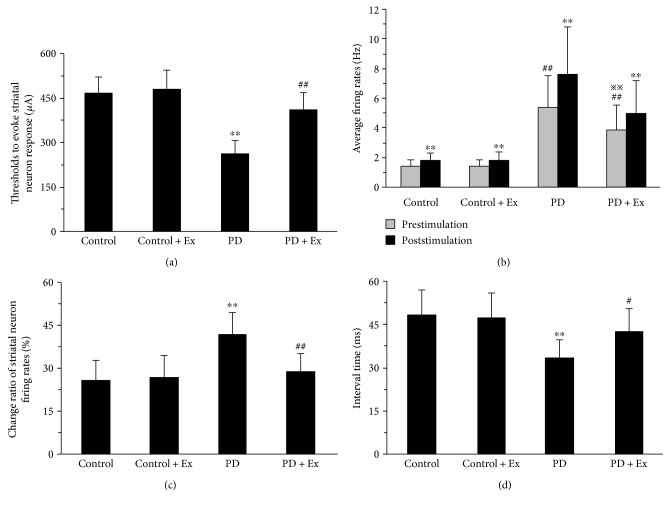
Effect of treadmill exercise on evoked electrical activity of striatal neurons in the rat model of Parkinson's disease. (a) The thresholds for evoking striatal neuron responses in rats. (b) Changes in average firing rates in striatal neurons after cortical stimulation. Analysis of spontaneous firing patterns in striatal neurons. (c) Change ratio of striatal neuron firing rates after stimulation. (d) The interval time of striatal neurons response after cortical stimulation. (a, c, d) Compared to the control groups, ^∗∗^*P* < 0.01. Compared to the PD groups, ^#^*P* < 0.05 and ^##^*P* < 0.01. (b) Compared to before cortical stimulation, ^∗∗^*P* < 0.01. Compare to the control groups, ^##^*P* < 0.01. Compared to the PD groups, ^※※^*P* < 0.05. Control: control group; control + Ex: control plus exercise group; PD: Parkinson's disease group; PD + Ex: Parkinson's disease plus exercise group.

A total of 94 silent striatal neurons, which could be activated by stimulating the primary motor cortex (M1), were recorded in rats. Among these, there were 23, 26, 20, and 25 striatal neurons that could be excited by stimulating the primary motor cortex in the control, control + Ex, PD, and PD + Ex groups, respectively. There was a significant decrease in the stimulus threshold of striatal neuron responses to cortical stimulation in PD rats (264 ± 41 *μ*A) as compared to control rats (468 ± 52 *μ*A) (*P* < 0.01). Moreover, the stimulus threshold of striatal neuron responses to stimulation was significantly increased in the PD + Ex group (412 ± 55 *μ*A) as compared to the PD rats group (*P* < 0.01), as shown in Figure 8(a).

There was a significant difference in striatal neuron firing rates before and after cortical stimulation at 1.5 mA in all rats (*P* < 0.01). The average firing rates after 60 s cortical stimulation significantly increased compared to those at the end of 60 s after cortical stimulation to all rats. The firing rate in the control group was increased by 26% (from 1.48 ± 0.34 Hz to 1.86 ± 0.42 Hz), and in the control + Ex group, it was increased by 27% (from 1.45 ± 0.39 Hz to 1.85 ± 0.55 Hz). In the PD group, this value was increased by 42% (from 5.41 ± 2.13 Hz to 7.67 ± 3.15 Hz). In the PD + Ex group, this variable was increased by 29% (from 3.88 ± 1.64 Hz to 5.02 ± 2.19 Hz). Therefore, the increased magnitude of firing rates in the PD group was significantly higher than that in the control group (*P* < 0.01). However, this variable in the PD + Ex group was significantly decreased as compared to that in the PD group (*P* < 0.05), as shown in Figures 8(b) and 8(c).

**Figure 9:**
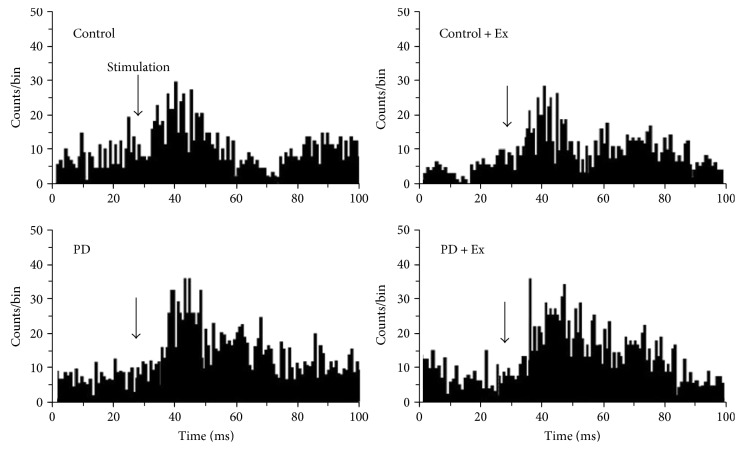
Comparison of evoked latency of striatal neurons after cortical stimulation. The latency was significantly decreased in the PD compared to the control group. Note that the latency in PD + Ex rats is significantly attenuated by treadmill exercise; exercise can reverse 6-OHDA-induced hyperactivity of the corticostriatal glutamatergic pathway. Control: control group; control + Ex: control plus exercise group; PD: Parkinson's disease group; PD + Ex: Parkinson's disease plus exercise group; 6-OHDA: 6-hydroxydopamine.

We found increased average firing rates of striatal neurons after cortical stimulation: 48.6 ± 8.3 ms, 33.5 ± 6.3 ms, and 42.7 ± 7.8 ms in the control, PD, and PD + Ex groups, respectively. The latency of the striatal neuron response, evoked by cortical stimulation, was significantly less in the PD than in the control counterparts (*P* < 0.01). We also observed higher latency in the PD + Ex rats than in the PD rats (*P* < 0.05). These findings suggest that the activity of the corticostriatal glutamatergic pathway was increased in PD rats compared to that in control rats. Importantly, these findings also indicate that treadmill exercise can effectively inhibit corticostriatal pathway excitability in PD rats, as shown in Figures 8(d) and 9.

### 3.5. Treadmill Exercise Decreases the Expression of Cav1.3 and p-CaMKII (Thr286) in the Striatum of PD Rats

**Figure 10:**
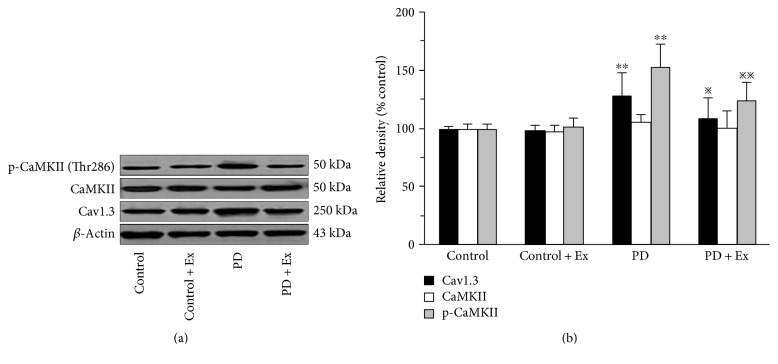
Protein expression of Cav1.3, CaMKII, and p-CaMKII in the rat's striatum. Compared to the control groups, ^∗∗^*P* < 0.01. Compared to the PD groups, ^※^*P* < 0.05 and ^※※^*P* < 0.01. Control: control group; Control + Ex: control plus exercise group; PD: Parkinson's disease group; PD + Ex: Parkinson's disease plus exercise group.

The results indicate that compared to control rats, the expression of Cav1.3 and p-CaMKII (Thr286) in the PD rats was significantly higher (*P* < 0.01, *P* < 0.01). Moreover, compared to PD rats, the expression of Cav1.3 and p-CaMKII (Thr286) in PD + Ex rats was significantly lower (*P* < 0.05, *P* < 0.01), as shown in Figure 10.

## 4. Discussion

Previous studies have shown that physical exercise improves motor performance and clinical symptoms of Parkinson's disease, mainly through changes in the motor circuit in the basal ganglia [14–16]. We adapted the cylinder test to measure motor function and electrophysiological techniques *in vivo* to measure the effect of treadmill exercise on corticostriatal pathway activity in PD rats. We demonstrated that treadmill exercise can improve motor function, reduce striatal neuron spontaneous firing activity, and inhibit hyperactivity of the corticostriatal glutamatergic pathway in PD rats. These findings suggest that the improvement in motor function caused by treadmill exercise may be related to reduced corticostriatal glutamatergic pathway activity in 6-OHDA-induced PD rats. To our knowledge, the effect of treadmill exercise on corticostriatal glutamatergic pathway activity in PD rats has not previously been investigated using electrophysiological techniques.

Striatal neurons are composed of MSNs, approximately 95% of the total, and intermediate neurons, including fast-spiking neurons (large), aspiny-like LANs (FS), and spiking LTS (low threshold) [17]. In the physiological state, most striatal neurons exhibited low firing rates, some were even silent, and the firing rates could change significantly by certain stimulations [17–19]. It is believed that most striatal neurons that spontaneously discharge are MSNs and that their firing rates are below 1 Hz [17]. Depolarization of the MSN membrane is considered to result from the input of cortical excitability, which is closely related to the release of glutamate from the cortex [20, 21]. Our previous studies demonstrated that striatal expression of D_2_R was significantly reduced in PD rats, which may lead to an increase of corticostriatal glutamatergic transmission due to a decrease in endocannabinoid system activity [21]. This may be one of the factors leading to enhanced spontaneous firing activity in striatal MSNs.

The primary motor cortex (M1) projects to the dorsal striatum, which may be involved in the consolidation and execution of nerve information-related movement [22, 23]. The loss of MSN dendritic spines in the dorsal striatum of PD rats can be inhibited by lesions in the primary motor cortex; the same effect can be produced by inhibiting the release of corticostriatal glutamate, which suggests that hyperactivity of the corticostriatal glutamatergic pathway is an important causative factor in changes in striatal neuron firing [24]. Striatal neuronal firing activity and corticostriatal pathway excitability can be enhanced by cortical stimulation [25]. We have also shown that striatal neuron firing activity was significantly enhanced in PD rats, and the cortical stimulation threshold to evoke striatal neuron responses and latency were significantly lower than in control rats, which further confirmed that corticostriatal glutamatergic activity was significantly enhanced in PD rats. After dopaminergic neurons were damaged by 6-OHDA, the dopamine denervation-induced inhibition to MSNs excitability was significantly decreased, while the transmission of excitatory neurotransmitters, such as glutamate and acetylcholine, was significantly enhanced [25, 26]. Therefore, MSNs transitioned from the quiescent state to the firing state, and this process was promoted by intracellular calcium ions, which may arise from glutamate acting on the related receptors or through activation of voltage-gated calcium channels [26–28]. The electrophysiological study suggested that the cortical stimulation threshold to evoke the striatal neuron responses in 6-OHDA-induced PD rats were decreased, which is consistent with the present study. We also found that evoked latency of striatal neurons was significantly shortened in 6-OHDA-induced PD rats; it was further indicated that the corticostriatal glutamatergic pathway was more highly activated in PD rats.

Furthermore, L-type voltage-gated Ca^2+^ channels are expressed in many cells of the central nervous system [29]. Among them, Cav1.3 subunits are abundant in MSNs. Although Cav1.3 is not involved in synaptic transmission, it is related to excitation-transcription coupling [30]. Several studies have shown that Ca^2+^/calmodulin-dependent protein kinase II (CaMKII) is closely related to this process [31, 32]. The intracellular Ca^2+^ level increases after Cav1.3 activation; CaMKII is autophosphorylated at Thr286, which is also accompanied by hyperactivity in the corticostriatal glutamatergic pathway in the dopamine-denervated striatum [32]. The present study also showed that 6-OHDA-induced PD rats had a significant increase in Cav1.3 and p-CaMKII (Thr286), but not CaMKII levels in the striatum compared to control rats. Therefore, increase in p-CaMKII (Thr286) may be closely associated with enhancement in the corticostriatal pathway, which mediates the biological effects of MSNs.

Numerous studies have indicated that exercise improves motor function and clinical symptoms in PD, possibly through neural plasticity changes in the basal ganglia [14–16]. Furthermore, those studies also showed that exercise could significantly modulate imbalances in DA and Glu, expression of related receptors and functional proteins in the striatum of PD rats, which may contribute to the improvement of corticostriatal glutamatergic transmission abnormalities [16, 33, 34]. Our previous studies have also shown that exercise can improve the loss of MSN dendritic spines in the striatum of PD rats. Most synaptic contacts, such as corticostriatal glutamatergic projection, occur at MSNs and the density of dendritic spines is affected by the activity of the glutamatergic pathway [9]. The decrease in MSN dendritic spines in PD rats is due, in part, to Ca^2+^ influx through Cav1.3. In the present study, the exercise intervention also reduced the expression of Cav1.3 and p-CaMKII (Thr286) in the striatum, which may be associated with exercise-induced inhibition of the corticostriatal glutamatergic pathway. Some studies have noted that dopamine depletion-induced hyperactivity of the corticostriatal glutamatergic pathway may play an important role in the pathogenesis of PD [3, 4]. Our results showed that 4 weeks of treadmill exercise may reduce the excitability of the corticostriatal pathway, inhibit synaptic transmission efficiency, and regulate MSN plasticity. These exercise-induced alterations are closely related to basal ganglia function. Therefore, we believe that alterations in the striatum may result in a reorganisation of basal ganglia motor control function in PD.

## 5. Summary and Conclusion

In summary, in the present study, we showed that treadmill exercise can regulate neuronal spontaneous firing activity in the striatum and inhibits the hyperactivity of the corticostriatal glutamatergic pathway in 6-OHDA-induced PD rats. Furthermore, we propose that these exercise-induced alterations, in particular, reduce corticostriatal glutamatergic pathway activity, which seem to be involved in improved Parkinson's disease-related motor dysfunction.

## Figures and Tables

**Table 1:** Comparison of the ratio of left forelimb touches to the total number of touches to the cylinder wall.

Groups	*n*	Number of wall touches of the left forelimb (%)		
		7th day	14th day	28th day
Control	50	50.15 ± 4.32	48.76 ± 4.73	49.82 ± 8.41
Control + Ex	50	52.43 ± 6.79	49.97 ± 6.54	51.43 ± 10.22
PD	52	27.36 ± 6.37^∗∗^	21.43 ± 6.36^∗∗^	15.35 ± 5.21^∗∗^
PD + Ex	44	31.49 ± 8.22^∗∗^	25.84 ± 5.87^∗∗^	26.24 ± 6.96^∗∗#^

Compared to the control groups, ^∗∗^*P* < 0.01. Compared to the Parkinson's disease (PD) groups, ^#^*P* < 0.05. Control: control group; control + Ex: control plus exercise group; PD: Parkinson's disease group; PD + Ex: Parkinson's disease plus exercise group.

**Table 2:** Distribution of spontaneous firing rate of striatal neurons in rats.

Group	*F* < 1 Hz		*F* = 1−10 Hz		*F* > 10 Hz	
	*f*($\bar{x}\pm s$)	Percentage	*f*($\bar{x}\pm s$)	Percentage	*f*($\bar{x}\pm s$)	Percentage
Control	0.36 ± 0.18	75.86%	4.31 ± 1.87	18.97%	14.79 ± 7.54	5.17%
Control + Ex	0.47 ± 0.23	62.67%	4.19 ± 1.56	24.0%	15.24 ± 5.28	13.33%
PD	0.53 ± 0.25	31.8%^∗∗^	4.97 ± 2.14	44.83%^∗∗^	20.58 ± 9.35	23.28%^∗∗^
PD + Ex	0.58 ± 0.27	45.92%^∗∗#^	3.98 ± 1.48	42.86%^∗∗^	18.32 ± 11.54	11.22%^#^

Compared to the control groups, ^∗∗^*P* < 0.01. Compared to the PD groups, ^#^*P* < 0.05. Control: control group; control + Ex: control plus exercise group; PD: Parkinson's disease group; PD + Ex: Parkinson's disease plus exercise group.
